# Robust Surface-Engineered Tape-Cast and Extrusion Methods to Fabricate Electrically-Conductive Poly(vinylidene fluoride)/Carbon Nanotube Filaments for Corrosion-Resistant 3D Printing Applications

**DOI:** 10.1038/s41598-019-45992-5

**Published:** 2019-07-03

**Authors:** Asma Almazrouei, Rahmat Agung Susantyoko, Chieh-Han Wu, Ibrahim Mustafa, Ayoob Alhammadi, Saif Almheiri

**Affiliations:** 1 Engineering Systems and Management, Khalifa University of Science and Technology, Masdar Institute, Masdar City, P.O. Box 54224, Abu Dhabi, United Arab Emirates; 2 Department of Mechanical Engineering, Khalifa University of Science and Technology, Masdar Institute, Masdar City, P.O. Box 54224, Abu Dhabi, United Arab Emirates; 3 Mohammed bin Rashid Al Maktoum Solar Park, Dubai Electricity & Water Authority (DEWA), Dubai, United Arab Emirates

**Keywords:** Electronic properties and materials, Materials science

## Abstract

We developed a poly(vinylidene fluoride)/carbon nanotube (PVDF-MWCNT) filament as a feed for printing of electrically-conductive and corrosion-resistant functional material by fused filament fabrication (FFF). Using an environment-friendly procedure to fabricate PVDF-MWCNT filament, we achieved the best reported electrical conductivity of printable PVDF-MWCNT filament of 28.5 S cm^−1^ (90 wt% PVDF and 10 wt% CNT). The PVDF-MWCNT filaments are chemically stable in acid, base, and salt solution, with no significant changes in electrical conductivity and mass of the filaments. Our processing method is robust and allow a uniform mixture of PVDF and CNT with a wide range of CNT percentage up to 99.9%. We demonstrated the printing of PVDF-MWCNT filaments to create 3D shapes; printed using a low-cost commercial consumer-grade FFF 3D printer. We found many adjustments of printer parameters are needed to print filament with CNT content >10 wt%, but easier printing for CNT content ≤10 wt%. Since this was due to printer limitation, we believed that PVDF-MWCNT with higher CNT percentage (to a certain limit) and larger electrical conductivity could be printed with a custom-built printer (for example stronger motor). PVDF-MWCNT filament shows higher electrical conductivity (28.5 S cm^−1^) than compressed composite (8.8 S cm^−1^) of the same 10 wt% of CNT, due to more alignment of CNT in the longitudinal direction of the extruded filament. Printable PVDF-MWCNT-Fe_2_O_3_ (with a functional additive of Fe_2_O_3_) showed higher electrical conductivity in the longitudinal direction at the filament core (42 S cm^−1^) compared to that in the longitudinal direction at the filament shell (0.43 S cm^−1^) for sample with composition of 60 wt% PVDF, 20 wt% CNT, and 20 wt% Fe_2_O_3_, due to extrusion skin effect with segregation of electrically insulating Fe_2_O_3_ at the shell surface of PVDF-MWCNT-Fe_2_O_3_.

## Introduction

Additive manufacturing or 3-dimension (3D) printing can produce rapid-prototyping of complex shapes^1–3^. There are numerous methods and applications of 3D printing such as in fluoropolymer based energetic material^4^, antennas^5^, carbon nanotube (CNT) yarn reinforcement^6^, electronic components^7,8^, fibre-reinforced polymeric materials^9^ and 3D printed parts by selective electroplating^10^. Specifically, fused filament fabrication (FFF), also known as fused deposition modelling (FDM), is the most economical and common additive manufacturing^1–3^. FFF works by forcing thermoplastic filament through a heated nozzle to form the desired 3D structures by mechanical movements in x, y and z directions^1,3^. Common filaments made from poly(lactic acid) (PLA) and acrylonitrile-butadiene-styrene (ABS) materials are mainly used as structural/aesthetic materials without additional functionality.

There are many applications in which the printed parts require corrosion resistance, chemical stability, and ability to withstand harsh environments. The potential for corrosion-resistant additive manufacturing has not been fully utilised. Table 1 listed example of applications where existing additive manufacturing cannot penetrate because of this issue. It is critical to overcome the limitation of existing materials used as filament feed in FFF.

**Table 1:** Example of applications where 3D printing faces corrosion challenges

No.	Applications	Cause of corrosion
1	Redox Flow Batteries	Sulfuric acid
2	Supercapacitors	Potassium hydroxide
3	Marine	Ions in seawater

Development of functional material for filaments enables rapid-prototyping of new models with unique properties, such as electrically conductive and corrosion resistance. Heikkinen *et al*.^11^ investigated the chemical resistance of non-conductive filaments in various solutions commonly used in semiconductor processing and found polypropylene as a promising material. To our knowledge, no report investigated the chemical stability of electrically conductive filaments in acidic, basic, or salt solution. Herein, polyvinylidene fluoride (PVDF) was chosen as the base thermoplastic as it is easy to process, and has room-temperature chemical corrosion resistance against sulfuric acid – a supporting electrolyte for vanadium redox flow batteries, and corrosion resistance against potassium hydroxide – an electrolyte for aqueous supercapacitors^12–16^. Moreover, PVDF can be used in piezoelectric applications^17^, for example: special grade PVDF-based co-polymers are commercially available for piezoelectric sensor and actuator.

A way to modify the electrical properties of thermoplastic polymers is by adding fillers. Carbon fillers such as CNT^18–20^, graphene^21^ and carbon black^22^ have been used to improve the functional, mechanical and electrical properties of polymers. Metal filler (for example silver nanowire, copper nanowire) can produce a highly conductive composite. However, metal nanostructures are prone to oxidation in air, which significantly decrease the electrical conductivity. In contrast, carbon-based filler (carbon black, graphene, carbon nanotube) are more resistant to oxidation in air, thus have better stability compared to metal fillers, and are also lighter than metal base fillers. Among the carbon-based fillers, we chose carbon nanotube since it has a high aspect ratio thus excellent conductivity according to percolation theory, lightweight, and is commercially available at a relatively low cost. PVDF-CNT composite (non-printable) have attracted scientists attention due to their exceptional properties such as high electrical conductivity^3,20,23^ up to 50.0 S cm^−1^ with 90% concentration of CNT to be used as a membrane^19^.

A lot of research has been done to improve the electrical conductivity of the PVDF-CNT composite (non-printable)^19,20,23,24^ but only a few focuses on producing high electrical conductivity of printable conductive filaments^3,21,22,25–29^. With regards to the printable polyvinylidene fluoride - multi wall carbon nanotube (PVDF-MWCNT) composite, Table 2 shows a summary of different filament compositions and their electrical conductivities.

**Table 2:** Different filaments compositions and their electrical conductivities.

Year	Filament Composite	Electrical Conductivity S cm^−1^	Applications	Reference
2017	CB/PP	2.0	Sensing applications/wearables	^22^
2017	Graphene/PLA	2.13	Lithium-ion anode	^21^
2017	CNT-Graphene/PBT	0.2	Filament for 3D printing	^25^
2017	MWCNT/PVDF	3.0 × 10^−2^	Electrically sense chemical vapours	^3^
2016	Carbon Black/PLA	0.34–0.67	Filament for 3D printing	^27^
2015	Graphene/ABS	1.05 × 10^−5^	Filament for 3D printing	^28^
2015	MWCNT/PLA	0.1–1.0	Liquid deposition modelling	^29^
2012	Carbon Black/PCL	0.11	3D printing of electronic sensors	^26^

CB = Carbon black, CNT = Carbon nanotubes, MWCNT = Multi-wall carbon nanotubes, PP = Polypropylene, PLA = Polylactic acid, PCL = Polycaprolactone, ABS = Acrylonitrile butadiene styrene.

**Figure 1:**
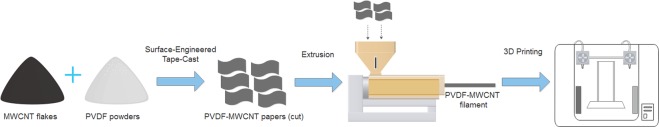
Illustration of the process fabrication of electrically conductive PVDF-MWCNT. Surface-engineered tape-cast can produce a uniform composition of PVDF-MWCNT papers with the broadest range of wt% MWCNT. Extrusion process converts the shape of paper to the filament of the desired diameter. 3D printing using Fused Filament Fabrication (FFF) of filaments using existing commercial FFF printer or custom-built FFF printer (for example stronger motor) for PVDF-MWCNT with ~10 wt% MWCNT or higher wt% MWCNT, respectively. Images are not to scale.

Figure 1 illustrates our processing method for fabrication of PVDF-MWCNT filaments for FFF. The first steps involve the mixing of PVDF powders and MWCNT flakes; and convert them to paper shape using Surface-Engineered Tape-Cast (SETC) technique^30–32^. We found that reported approach in the literature (called solution-cast) resulted in the uniform composition of PVDF-MWCNT, but was limited to ~15 wt% CNT due to high viscosity limitation during the solution-cast process^3^. We believe that there is a need to incorporate more CNT in the PVDF-MWCNT composite, for example, to increase the electrical conductivity further. Thus, a composite fabrication process that is not limited in the viscosity is needed. Therefore, we proposed the SETC approach which can result in a relatively uniform composition of PVDF-MWCNT paper at wide composition range, from 0.1% to 99.9% MWCNT. In the second steps, the extrusion of the PVDF-MWCNT paper was done, resulting in filaments to be 3D-printed. The third steps of FFF can be done using commercial FFF printer (as demonstrated in this paper) or using a custom-built FFF printer with a stronger motor to allow printing of filaments with a large percentage of MWCNT. Note that using our method (see Fig. 1), there is no viscosity limitation in the first step, however melt-viscosity of the composite plays a significant role in the second and third steps. In contrast, all steps in the previous report were limited by the viscosity which hinder the improvement of the electrical conductivity^3^.

To our knowledge, there is no 3D printing’s filament that has both properties of high electrical conductivity (>10 S cm^−1^) and excellent chemical resistance, e.g., in sulfuric acid (H_2_SO_4_), potassium hydroxide (KOH), or sodium chloride (NaCl) solution. Herein, we developed a printable PVDF-MWCNT composite as a conductive and corrosion resistant filament for FFF additive manufacturing. We performed the electrical and physical characterisations of the resulting composite. We also investigated the effect of iron oxide (Fe_2_O_3_) addition to the electrical conductivity.

## Experimental Methods

### Materials

Poly(vinylidene fluoride) of product number 44080 (denoted as P1), with specification according to datasheet: melting point of 155–160 °C, and relatively high melt viscosity of 29.4 kPoise (tested at 232 °C, 100 s^−1^) was purchased from Alfa Aesar. Kynar^®^ 721 poly(vinylidene fluoride) (denoted as P2) powder with datasheet specification: melting point of 165–172 °C, melt flow of 5 to 29 g over 10 min at 230 °C, and relatively low melt viscosity of 5 to 12 kPoise (tested at 230 °C, 100 s^−1^, ASTM D3835) was provided from ARKEMA. KYNAR^®^ 721 is a special grade PVDF and a registered trademark from ARKEMA. MWCNT flakes (denoted as C1) were supplied from Applied Nanostructured Solutions (ANS), Baltimore, which has a strand length of 25 to 140 µm, an outer diameter of 29 to 40 nm, aspect ratio of 625 to 4827, with features of pre-alignment and cross-linking^33,34^. Multi-wall carbon nanotubes with a strand length of 3 to 30 µm, an outer diameter of 13 to 18 nm, aspect ratio of 166 to 2307, and a purity >99 wt% (denoted as C2) were supplied from Cheaptubes. Iron oxide (Fe_2_O_3_) nanoparticles (particle size < 50 nm) was sourced from Sigma-Aldrich with the product number of 544884. Ethanol solvent of ≥99.8% purity was obtained from Sigma-Aldrich. Deionised water (resistivity ≥18.2 MΩ cm) was generated using Purite Select Fusion Deionised Water Purification System. All materials were used as received.

### Processing

#### Preparation of the composite powder

MWCNT flakes (0.4 gram) were dispersed in 20 ml deionised water and 20 ml ethanol and ground in a mortar for 2 minutes. Then the mixture was transferred to the 1 L glass beaker, PVDF powders (3.6 grams for 90 wt%PVDF-10 wt%MWCNT), 80 ml deionised water and 80 ml ethanol were subsequently added. The solution was exposed to ultrasonication and mechanical stirring with a VCX 750 Ultrasonic Processor (Sonic, USA) and Advanced Hotplate Stirrer (VWR, USA) for 2 min at 500 rpm and 8 min at 800 rpm while the amplitude of sonicator was set at 40%. Then the mixture was tape-casted on top of copper foil (matt-side facing up)^30–32^ with 5 mm gap and dried in a convection oven (Binder Forced Convection Oven FD 53) at a temperature of 120 °C for 1 hour. After drying, the composite sheet was peeled off from copper sheet and cut into small pieces. The same process was repeated for the PVDF-MWCNT-Fe_2_O_3_ sample, but with the addition of Fe_2_O_3_, in which MWCNT and Fe_2_O_3_ were ground for 2 minutes before adding PVDF; the rest procedures were similar.

#### Preparation of the compressed composite sample

MWCNT–PVDF composite powders were made using different sonication time (10, 30, and 60 minutes; at the same amplitude of 40%) and different sonication amplitude (40, 60, and 80%; at the same timing of 10 minutes). After cast and dried, then, to make the compressed composite, they were placed in between two stainless-steel sheet and compressed using Carver Auto CH-NE compressing machine using 500 kg, over 6-inch x 6-inch plate, at 170 °C for 10 minutes. After that, the sample and stainless-steel sheets were quickly moved out from the machine, followed by a regular room cooling. The resulting compressed composite sample was then cut as a square in 2 cm length × 2 cm width, for electrical conductivity analysis using LakeShore Hall effect measurement system (HMS, model 7607).

#### Preparation of the extruded composite filament

As the extrusion machine requires a large quantity of composite powders, plenty of composite powders were prepared by following Section 2.2.1, except 250 ml deionised water and 250 ml ethanol as solvents were used; as well as 1 gram of MWCNT + 19 grams of PVDF, 1 gram of MWCNT + 9 grams of PVDF, and 1 gram of MWCNT + 5.67 grams of PVDF, were used for 95 wt%PVDF-5 wt%MWCNT, 90 wt%PVDF-10 wt%MWCNT, and 85 wt%PVDF-15 wt%MWCNT compositions, respectively. The procedures were repeated until at least 20 grams of dried composite powders were obtained for each composition.

PVDF-MWCNT and PVDF-MWCNT-Fe_2_O_3_ powder composites were extruded using Noztek Pro high-temperature extruder (17.5 Nm motor) to get the filaments of 3 mm diameter. Many tests were done to optimise the extruding temperature of the composite filament (190–300 °C). We found an optimised extruding temperature of 230–240 °C. We did not control the cooling method after extrusion. The cooling after extrusion was by the built-in small fan and regular room cooling. The filament was then tested inside the BCN3D Sigma Release 2017 printer.

#### Preparation of 3D-printed objects

BCN3D Sigma Release 2017 printer is an FFF printer used to print from a feed of solid filaments. Different ranges of printing temperatures were used to optimise the printing temperature and to study the effect of temperature on electrical conductivity (200–300 °C). Heated bed was used in a range of 100 to 200 °C. Since the standard heated bed is limited to 115 °C, we modified the heated bed of BCN3D Sigma Release 2017 printer: by adding glass plate, an external silicone heating sheet with adjustable temperature control, and thermal insulation to prevent damaging of the printer stage. This adjustment increased the temperature capability of the heated bed up to 300 °C. The nozzle was made from stainless steel with a diameter of 2 mm. The .stl file of the object was designed using Blender software. The object/model was 3D printed at a nozzle temperature of 250 °C by using default speed of 250 mm min^−1^, x/y speed at 300 mm min^−1^ and z speed at 900 mm min^−1^. The heated bed was set at 200 °C. Supplementary video 1 shows filament successfully passed through the nozzle of the 3D printer; as well as the printing of a model of disk shape. We did not vary the cooling method of the printed PVDF-MWCNT after passing through nozzle. The cooling after 3D printing was by the built-in small fan and regular room cooling.

### Characterisations

A digital multimeter (Keysight 34465) with four-probe configuration was used to test the electrical conductivity of filament and printed samples, at room temperature, and without silver paste. LakeShore Hall effect measurement system (HMS, model 7607), was used to analyse the electronic transport properties of the compressed composite samples using van der Pauw configuration, at room temperature, and with silver paste at the interface of sample and probe for contact-resistance minimisation.

Thermal characterisation was performed using thermal gravimetric analysis (TGA) using NETZSH TGA to study the mass changes with increasing temperature. The TGA was used to characterise the purity and thermal stability of MWCNT under an oxygen atmosphere of 20 ml min^−1^ flow and temperature from 30 to 1000 °C. Physical characterisation was done using scanning electron microscopy of Nova NanoSEM 650. Atomic force microscopy (AFM) was used to study the topography map of our filaments using Witec Alpha 300RAS, with non-contact mode and 42 Nm^−1^ tip resonance frequency.

## Results and Discussion

### Effect of processing parameters to electrical conductivity

It is known that the electrical conductivity of graphite can reach 1000 S cm^−1^ but when the polymer is added the conductivity can drop to 10 S cm^−1^ ^35^. Formulation of the composite mixture to improve the filament’s electrical conductivity was done by testing different MWCNT type, PVDF type, MWCNT concentration, sonication time, and printing temperature as the following:

#### The effect of CNT and PVDF types

**Figure 2:**
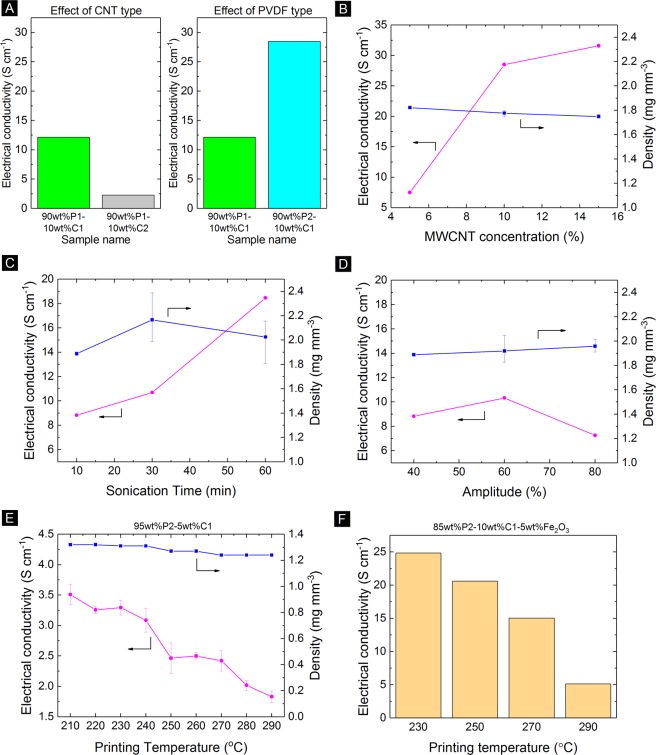
A study of different factors that affect the electrical conductivity of (**A**,**B**) poly(vinylidene fluoride)-multiwall carbon nanotube (PVDF-MWCNT) extruded filaments: (A) the effect of MWCNT type (C1 and C2) and PVDF type (P1 and P2) to the electrical conductivity of PVDF-MWCNT extruded filaments, (**B**) the effect of MWCNT (C1) concentration to the electrical conductivity of 90 wt%P2-10 wt%C1 extruded filament; (**C**–**D**) MWCNT compressed composite: (**C**) the effect of sonication time and (**D**) sonication amplitude to the electrical conductivity of 90 wt%P2-10 wt%C1 compressed composite. (**E**-**F**) The effect of printing temperature on the electrical conductivity using (E) PVDF-MWCNT filament of 95 wt%P2-5 wt%C1 and (F) PVDF-MWCNT-Additive filament of 85 wt%P2-10 wt%C1-5wt%Fe_2_O_3_.

First, we varied only the CNT type. Two filaments with the same type of PVDF (P1) were tested with two types of carbon nanotubes (C1 and C2), referred to 90 wt%P1–10 wt%C1 and 90 wt%P1–10 wt%C2, to study the change in the electrical conductivity. Figure 2A shows that 90 wt%P1-10 wt%C1 has a higher electrical conductivity (12.14 S cm^−1^) compared to 90 wt%P1-10 wt%C2 (2.29 S cm^−1^) by one order of magnitude due to the relatively long wires of 25 to 140 µm, cross-linking and pre-alignment of CNT (C1)^33,34^ in 90 wt%P1-10 wt%C1. In contrast, CNT (C2) has a relatively short length of 3 to 30 µm, no crosslinking and pre-alignment.

Next, we varied only the PVDF type. Using the same type of MWCNT (C1) we conducted another experiment with different type of PVDF (P1 and P2), referred to 90 wt%P1-10 wt%C1 and 90 wt%P2-10 wt%C1. We noticed that the electrical conductivity of 90 wt%P2-10 wt%C1 reaches 28.5 S cm^−1^ while 90 wt%P1-10 wt%C1 reached a lower value of 12.14 S cm^−1^, see Fig. 2A. This result is because PVDF (P2) has a relatively low melt viscosity of 5 to 12 kPoise, as compared to the relatively high melt viscosity of 29.4 kPoise of PVDF (P1). Thus, MWCNT with a length of 25 to 140 µm (C1) and PVDF powder with a melt viscosity of 5 to 12 kPoise (P2) was chosen to proceed with further experiments.

It is noted that electrostatic dissipation (EDS) requires electrical conductivity of at least 10^−6^ to 10^−2^ S cm^−1^ while electromagnetic interference (EMI) shielding requires electrical conductivity >10^−2^ S cm^−1^ ^36^. In all cases, our PVDF-MWCNT composite able to meet EDS and EMI shielding requirements.

#### The effect of MWCNT concentration

As the concentration of MWCNT (C1) increased (5, 10 and 15%), the electrical conductivity increased too (9.4, 28.5 and 29.5 S cm^−1^), for sample 95 wt%P2-5 wt%C1, 90 wt%P2-10 wt%C1, and 85 wt%P2-15 wt%C1, respectively, see Fig. 2B. Similar experimental results were also reported by E. Backes^37^, V. Choudhary^38^ and F. Du^39^. This trend is because of easier percolation with more concentration of MWCNT in the polymer composite. Compared to works by Balberg^40^, Celzard^41^, Kharchenko^42^, and Rahatekar^43^, our C1 filler had a large aspect ratio of 625 to 4827 compared to the maximum aspect ratio of 75, 200, 1000, and 200 in the reference^40^, reference^41^, reference^42^, and reference^43^, respectively. The larger the aspect ratio, the smaller the critical concentration (concentration where transition of electrically-insulating to electrically-conducting behaviour occurs)^40–43^. As shown from the electrical conductivity values, our tested composition of 5, 10 and 15 wt% C1 are in the electrically-conducting region; where the filler concentration was higher than the critical concentration. Comparison of the electrical conductivity value at similar aspect ratio and filler concentration was not possible due to scope limitation of the previous works^40–43^. Figure 2B shows that the density was similar; within the range of 1.75 to 1.82 mg mm^−3^. We observed that the filaments were easily printable up to 10% MWCNT concentration, but adjustments to the printer parameters were needed at 15% MWCNT concentration. With a higher concentration of MWCNT concentration of >15%, the extrusion becomes more challenging due to extruder’s motor limitation, due to the high viscosity of the heated filaments. We observed that the 15% MWCNT samples could be bent and did not break during winding. However, filament breaking during winding in a bobbin could be a problem for MWCNT concentration of >15%. This is worth for investigation in the future works.

The 3D printing technique of PVDF-MWCNT and PVDF-MWCNT-Additive (such as Fe_2_O_3_ additive) are promising for the particular application and tailored for printing of large part (with x and y resolution of at least 2 mm). Note that due to the relatively high viscosity at typical printing temperature, the nozzle opening diameter needs to be large (1.5 mm or more)^26^, thus this technique is only compatible to print large-size parts. The 3D printing of large parts is promising for economic benefit compared to the conventional subtractive method of large parts^44^.

#### The effect of sonication time and amplitude

In this section, we have another set of experiments using compressed composite instead of extruded composite. The motivation of using compressed composite is to investigate if there is any anisotropy effect of the electrical conductivity compared with using the extrusion process. For the sonication time, it was reported by G. Faiella^45,46^ that increasing sonication times increase the percolation threshold volume fraction so the electrical conductivity. Moreover, sonication improves the dispersion and mechanical properties of nanocomposite^47^. As the sonication time increases from 10 to 60 min, the conductivity increases from 8.8 to 18.5 S cm^−1^ as shown in Fig. 2C.

When the amplitude of the sonicator was increased from 40 to 60%, the electrical conductivity increased from 8.8 to 10.3 S cm^−1^, respectively, presumably due to better dispersion of MWCNT and PVDF in the solution^47^. However, as we went further to 80% amplitude, the electrical conductivity dropped to 7.3 S cm^−1^ (as shown in Fig. 2D). This change might be due to shortening wires length of MWCNTs when too much power of the ultrasonic wave was delivered. The results provide valuable insights into the effect of sonication to the electrical conductivity of CNT and suggest an optimum value of the sonication amplitude and time. Mustafa *et al*^13^. reported that when 40% amplitude was used, the electrical conductivity of the CNT sheet decreased with increasing sonication time. We attribute the difference in the trend due to the damping mechanism of PVDF acting as a protective layer for the CNT during sonication. However, when the sonication amplitude is high (80%), the effect diminished, and electrical conductivity dropped.

Anisotropy effect of the electrical conductivity was observed when the extrusion process is used instead of the compression process. At the same composition of 90 wt%P2-10 wt%C1, same sonication time of 10 min, and same amplitude of 40%, we observed that compressed composite has a lower electrical conductivity of 8.8 S cm^−1^ compared to the extruded filament of 28.5 S cm^−1^. The reason for this could be due to the higher alignment of CNT in the longitudinal direction of the extruded filament. The extrusion process forced the CNT to align parallel with the direction of extrusion. The evidence of alignment is shown in section 3.3 of scanning electron microscopy. Although the compressed composite samples were processed at a relatively low temperature of 170 °C, we used a relatively long time (10 minutes) for the PVDF to melt and join with the MWCNT filler. Optimisation of processing temperature for the compressed composite samples is very interesting for future works.

#### The effect of printing temperature on electrical conductivity

To our knowledge, no report talks about the effect of printing temperature on electrical conductivity at room temperature. Y. Zeng^48^ reported that the electrical resistivity increased sharply with increasing the temperature/the electrical conductivity dropped with higher temperature mainly because of the breakdown of interconnection of the CNT network. E. Bilotti^49^ reported lower resistivity at 240 °C compared to 200 °C for TPU/CNT/CB filament. The decrease in conductivity is partially due to the decrease in the density of carriers^50^. The increase in speed of printing the filament could also decrease the electrical conductivity as the CNT network is destroyed^49^. Figure 2E shows that as the temperature of printing of 95 wt%P2-5 wt%C1 was increased from 210 to 290 °C, the electrical conductivity of the printed PVDF-MWCNT decreased from 3.5 to 1.8 S cm^−1^. The same decreasing trend was also observed for PVDF-MWCNT filament with 5% Fe_2_O_3_ (sample name = 85 wt%P2-10 wt%C1-5 wt%Fe_2_O_3_); as the temperature increased from 230 °C to 290 °C, the electrical conductivity dropped from 24.8 to 5.14 S cm^−1^, see Fig. 2F. The optimised printing temperature of around 250 to 270 °C was a trade-off of easiness to print (less viscosity at higher printing temperature) and electrical conductivity (more electrical conductivity at lower printing temperature). Future study using multiple characterisation techniques are needed to understand the effect of temperature to the MWCNT distribution in the composite.

### Thermal Characterisation

**Figure 3:**
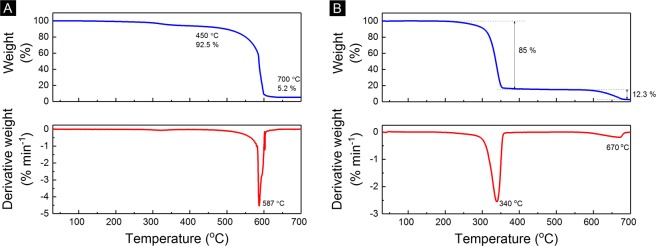
(**A**) Thermal gravimetric analysis (TGA) curve of MWCNT (C1) and its first derivative weight. (**B**) TGA curve of PVDF-MWCNT (P2-C1) filament and its first derivative weight.

Thermal gravimetric analysis (TGA) in a pure oxygen atmosphere was performed on as received MWCNT. Figure 3 shows that the purity of MWCNT (C1) was 87.5% with 7.5% carbonaceous impurities after 400 °C and 5% non-carbonaceous impurities (e.g., metal catalyst) after 600 °C. The decomposition temperature of MWCNT (C1) was about 587 °C, which was determined by the peak oxidisation temperature (point of maximum weight loss) as shown from the differential of the weight in Fig. 3A.

Another TGA test was done on the 90 wt%P2-10 wt%C1 filament to study the mass change as temperature increase up to 700 °C under air at a heating rate of 5.0 K min^−1^. At 240 °C, the composite started to decompose due to the polymer and the degradation at 340 °C with a total loss of around 85% after 400 °C, see Fig. 3B. The MWCNT content was 12.3%, calculated from the weight difference at 400 and 700 °C. The remaining 2.7% at 700 °C was due to non-carbonaceous impurities in the MWCNT. The derivative weight peak was 340 °C, meaning that the polymer can withstand the heat of less than this temperature. The next derivative peak was 670 °C, because of the CNT content.

### Morphological structure studies by Scanning Electron Microscopy (SEM)

#### SEM of PVDF-MWCNT composite powder

**Figure 4:**
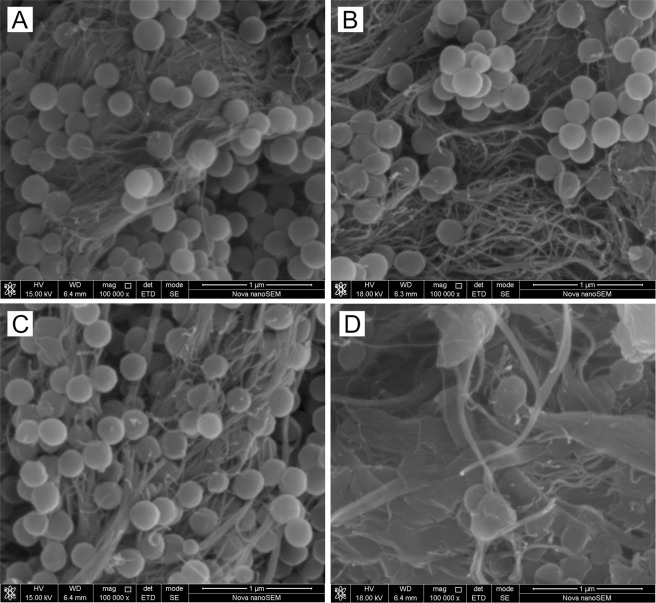
Scanning electron microscopy (SEM) shows the structure change of 95 wt%P2-5 wt%C1 composite powder (before extrusion) at 100,000 times magnification (**A**) without ball-milling, and, (**B**–**D**) after ball-milling for (**B**) 10 minutes, (**C**) 30 minutes, and (**D**) 60 minutes.

Figure 4 shows the effect of ball milling of 95 wt%P2-5 wt%C1 to the structure at different magnifications. The PVDF (P2) diameter before ball-milling and after 30 minutes was measurable as circular shapes (250–300 nm). After ball-milling for 1 hour, the PVDF diameter was not measurable any more, but the nanocomposite becomes more homogenous after the ball-milling. The literature reported nanoparticles were produced after using ball milling for multi-wall carbon nanotubes (MWCNTs)^51^ and the MWCNT length decreases with increasing the milling time^52^ and speed^53^. In the ball-mill samples, the MWCNT became more entangled, and the polymers became more attached to MWCNT; compared to without ball milling. While ball mill could break the agglomeration of the PVDF spheres, long timing of ball mill could lead to a sufficient temperature increase inside the jar which soften or melt the PVDF spheres to improve the attachment of PVDF and MWCNT. Although we did not measure the increase of the temperature inside the jar, previous reports showed that ball milling can introduce a significant increase in the temperature inside the jar, for example 60 to 600 °C, depending on many factors^54^. After ball-milling for 60 minutes, the samples were charging which means it becomes less conductive and thin gold coating was needed to get better SEM images.

The morphology – before extrusion – of 90 wt%P2-10 wt%C1 (Supplementary Fig. S1) is similar as that of the 95 wt%P2-5 wt%C1 (Fig. 4A). The PVDF polymer spheres were more dispersed with the treatment of 10-minute ball milling, compared to without ball mill for the sample of 90 wt%P2-10 wt%C1 composite powder (before extrusion) (see Supplementary Fig. S1).

#### SEM of PVDF-MWCNT filament and 3D printed sample

**Figure 5:**
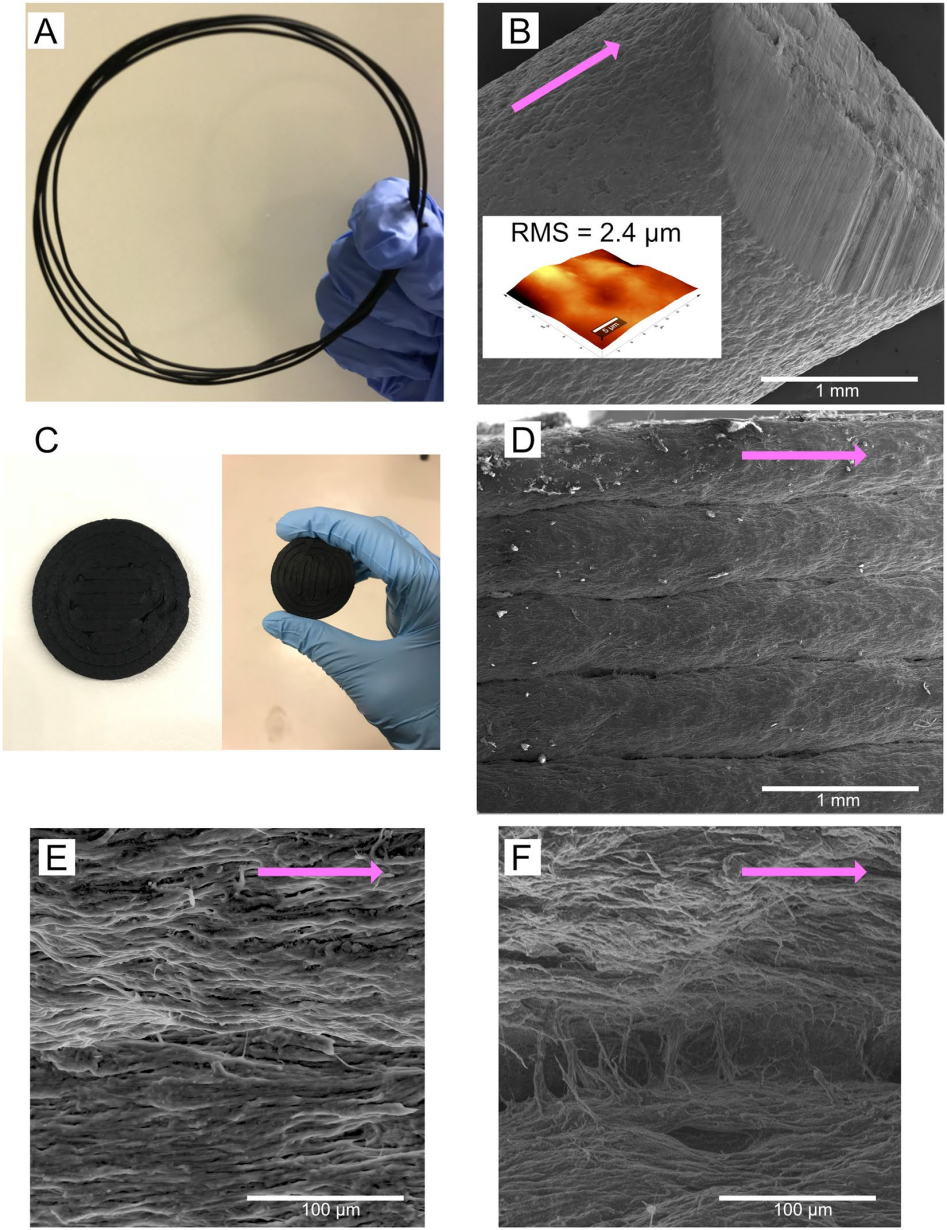
(**A**) A visual image of the extruded 95 wt%P2-5 wt%C1 filament (**B**) Scanning electron microscopy (SEM) image of the cross-section of the 95 wt%P2-5 wt%C1 filament (inset = atomic force microscopy images shows root mean square roughness of 2.4 µm). (**C**) 3D printed sample disk from the printing of 95 wt%P2-5 wt%C1 filament. SEM images show (**D**) the layers of the sample from the top view, (**E**) high magnification of the layer showed directionality of the CNT strands parallel to the extrusion direction, and (**F**) high magnification of the connection between each layer.

The printing was done with 95 wt%P2-5 wt%C1 using BCN3D Sigma Release 2017 printer with 2 mm nozzle diameter. Figure 5A shows the conductive filaments used as a feed for 3D printing. Supplementary Fig. S2 shows the 90 wt%P2-10 wt%C1 conductive filaments. Figure 5B shows the SEM image of the conductive filament with the inset shows the AFM mapping of the surface with a root mean square roughness of 2.4 µm. The nozzle printing temperature was set at 250 to 270 °C, while bed temperature was set at 200 °C. We were able to print a disk shape of 15 mm diameter and 2 mm thickness, see Fig. 5C. The printed layer thickness from 2 mm nozzle showed to be equal to 2.4 ± 0.1 mm. Figure 5D shows the top view of the printed disk. It is observed that the CNT is aligned parallel to the direction of the printer nozzle’s extrusion, as seen in Fig. 5E. Figure 5F shows the connection between layers; the printed layers were linked to each other to form the solid conductive disk.

### Chemical stability of electrically-conductive filaments

**Figure 6:**
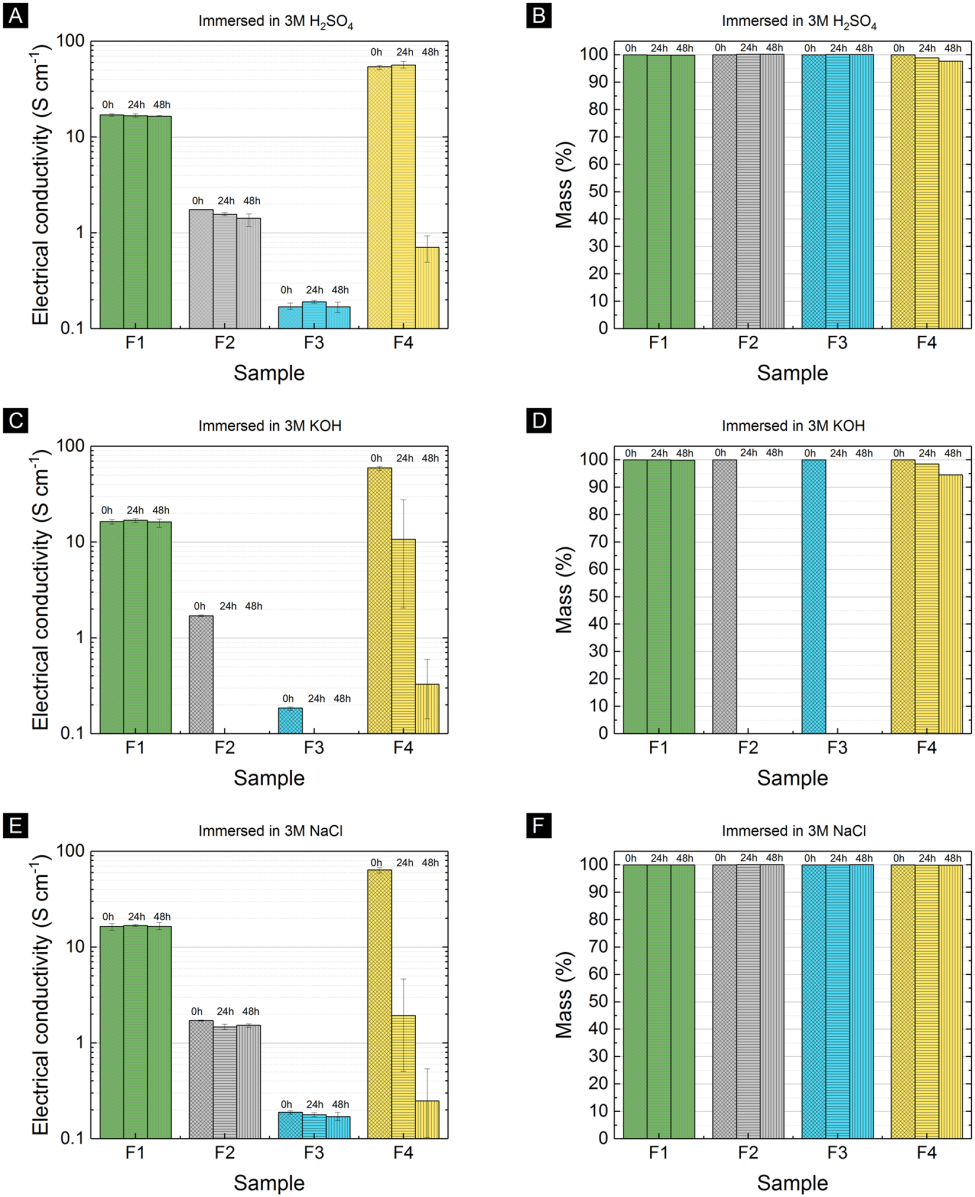
The electrical conductivity and mass change of our filament (sample F1) compared to commercial filaments (sample F2, F3 and F4) after immersed at different aqueous media: (**A**,**B**) 3M H_2_SO_4_, (**C**,**D**) 3M KOH, and (**E**,**F**) 3M NaCl solution.

The chemical stability of 4 different types of electrically conductive filaments is shown in Fig. 6. The samples are PVDF-MWCNT filament of 90 wt%P2-10 wt%C1 (extruded at 280 °C) (this work), conductive graphene PLA based filament (blackmagic3d.com), conductive PLA filament (proto-pasta.com), and conductive metal-filler-based proprietary filament (multi3dllc.com), denoted as F1, F2, F3, and F4, respectively. Sample F4 comprises of copper and silver fillers, according to energy-dispersive x-ray spectroscopy (see Supplementary Fig. S3). The mass and electrical conductivity of filaments were measured before immersion. The electrical conductivity – before immersion – of F1 was in average of 16.6 S cm^−1^, slightly lower than the 28.5 S cm^−1^ of same sample composition of 90 wt%P2-10 wt%C1 in Fig. 2A, due to different batch (in this section 3.4, the extrusion was performed at 280 °C, not at optimised temperature of 230–240 °C). Then, filaments were immersed each in a different solution of 3M H_2_SO_4_, 3M KOH, and 3M NaCl, with stirring bar set at 300 rpm, for 24 hours and 48 hours. It was then followed by rinsing in DI water and drying at room temperature. The mass and electrical conductivity of filaments were also measured after 24 hours, and 48 hours of immersion.

We confirmed the chemical compatibility of PVDF-MWCNT in acid, base, and salt media, see Fig. 6. Figure 6A shows filaments with carbon-based filler (F1, F2, and F3) have relatively stable electrical conductivity and mass after 48 hours of immersion in 3M H_2_SO_4_. Among filaments with carbon-based filler, the sample F1 has the highest electrical conductivity. On the other hand, sample F4 retain only 1.3% of electrical conductivity and 97.7% mass, after 48 hours of immersion in 3M H_2_SO_4_. We attributed this loss due to the incompatibility of the metal-based filler of sample F4 in the acidic media.

Figure 6B shows the stability of our filament (F1) compared to other commercial filaments in 3M KOH solution. The electrical conductivity and mass of sample F1 were retained after 48 hours of immersion in 3M KOH. Sample F2 and F3 were disintegrated after 24 hours of immersion in 3M KOH, due to decomposition of the PLA polymer. Sample F4 retained only 0.6% of electrical conductivity and 94.6% of mass, due to the incompatibility of the metal-based filler of sample F4 in the base media.

Figure 6C shows the relatively stable mass of all samples. Carbon-based filler filaments (F1, F2, and F3) have relatively stable electrical conductivity after 48 hours of immersion in 3M NaCl. However, sample F4 retained only 0.4% electrical conductivity, after 48 hours of immersion in 3M NaCl, due to corrosion of metal-based filler of sample F4 in the salt media.

### Anisotropy of electrical conductivity of filament and 3D printed PVDF-MWCNT-Fe_2_O_3_

We investigated the effect of Fe_2_O_3_ addition to the electrical conductivity. The motivation was to insert a functional additive, Fe_2_O_3_ as an example, which has other useful properties. Despite an electrically insulating material, the Fe_2_O_3_ has magnetic property and/or electrochemical property, which could be applied in magnetic-based device and/or battery application. The average electrical conductivity for the 75 wt%P2-5 wt%C1–20 wt%Fe_2_O_3_ filament was 4.05 S cm^−1^, while 85 wt%P2-10 wt%C1–5 wt%Fe_2_O_3_ filament was 25.01 S cm^−1^ due to the increase in MWCNT concentration. When each concentration of MWCNT and Fe_2_O_3_ increased to 20 wt%, as in sample 60 wt%P2-20 wt%C1–20 wt%Fe_2_O_3_, the average electrical conductivity increased to 42.0 S cm^−1^, see Table 3.

**Table 3:** Filaments of PVDF–MWCNT–Fe2O3.

Sample name	Mass of MWCNT (g)	Mass of Fe_2_O_3_ (g)	Mass of PVDF (g)	Sonication Power (kJ)	Electrical conductivity (S cm^−1^)
75 wt%P2-5 wt%C1–20 wt%Fe_2_O_3_	1	4	15	19.3	4.05
85 wt%P2-10 wt%C1–5 wt%Fe_2_O_3_	2	1	17	19.0	25.01
60 wt%P2-20 wt%C1–20 wt%Fe_2_O_3_	4	4	12	19.2	42.0

The temperature of extrusion is the main factor determining the shearing and viscosity of materials^50^. The filament was extruded efficiently at a temperature of 230 ± 10 °C. The 3D printing was tested in a range of 250 to 290 °C nozzle temperature, and the heated bed was covered with a tape and PVDF glue to enhance the adhesion of the printed object. The bed temperature was maintained at 250 °C to avoid solidifying of the printed object. We observed that the bed requires high adhesion and rough surface, and, the printing speed, directions in x-y-z and layers affected the outputs from the 3D printer. The 3D printing allowed having different infill ranges, sizes, and different electrical and mechanical properties.

**Figure 7:**
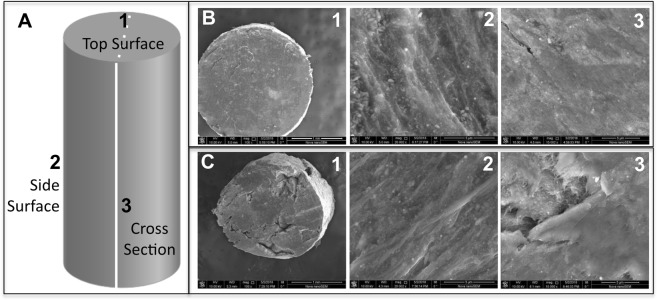
(**A**) Illustration of the PVDF-MWCNT-Fe_2_O_3_ filament/printed sample. (**B**,**C**) Scanning electron microscopy (SEM) images of 60 wt%P2-20 wt%C1–20 wt%Fe_2_O_3_ filament is showing the three surfaces of (**B**) the filament, and (**C**) printed sample.

Printable 60 wt%P2-20 wt%C1–20 wt%Fe_2_O_3_ shows higher electrical conductivity in the longitudinal direction at the filament core (42 S cm^−1^) compared to that in the longitudinal direction at the filament shell (0.43 S cm^−1^). SEM imaging was done on three surfaces of the filament and 3D printed samples of PVDF-MWCNT-Fe_2_O_3_ to check the morphology and relate with electrical conductivity. Figure 7 shows there was more concentration of PVDF on surface 2 than on other surfaces which cause to have lower electrical conductivity on shell/surface 2 (0.43 S cm^−1^) compared to core/surface 1 (42 S cm^−1^). Also, we found that MWCNT strands were aligned along the extrusion direction during printing, which also explained the high conductivity on surface 1. The interface/surface 3 has less PVDF than surface 2, and more CNT oriented in one direction, which is the same direction of the extrusion and printing. Compared to the feed filament, the printed sample has more empty spaces and disorientation, which might be due to the high force of the nozzle’s printer to extrude compared to the slow extrusion when making the feed filament, see Fig. 7. Whereas in this section, we have provided a preliminary observation of the anisotropy of the electrical conductivity in the PVDF-MWCNT-Fe_2_O_3_ system, we think that future works need to be done to systematically understand the effect of various processing parameters such the extrusion speed, extrusion force, etc. to the electrical conductivity and anisotropy of the electrical conductivity.

## Conclusions

We reported conductive filament of PVDF-MWCNT and PVDF-MWCNT-Fe_2_O_3_ made using non-toxic solvent and environmental-friendly process (usage of water and ethanol solvents instead of toxic solvents of N,N-dimethylformamide and N-methyl-2-pyrrolidone; low cost of chemical waste disposal, and low energy consumption of the dispersion process). PVDF-MWCNT filament (10 wt% MWCNT) showed an excellent electrical conductivity of 28.5 S cm^−1^ along the direction parallel to the extrusion direction. PVDF-MWCNT-Fe_2_O_3_ filament (20 wt% CNT, 20 wt% Fe_2_O_3_) showed higher electrical conductivity in the longitudinal direction at the filament core (42 S cm^−1^) compared to that in the longitudinal direction at the filament shell (0.43 S cm^−1^). To our knowledge, the produced filaments have the highest electrical conductivity, which is vital to make a functional printed material using fused filament fabrication. We also confirmed the chemical stability of PVDF-MWCNT filaments in 3M H_2_SO_4_, 3M KOH, and 3M NaCl with no significant changes in electrical conductivity and mass after 48 hours of immersion. The PVDF-MWCNT filaments are promising for 3D printing of electrically conductive functional material for corrosion-prone applications. Our robust SETC method allowed preparation of uniform sheets of PVDF and MWCNT mixture, with a wide range of MWCNT percentage (up to MWCNT percentage up to 99.9%).

## Supplementary information

Supplementary Information

Supplementary Video

## Data Availability

The datasets generated during and/or analysed during the current study are available from the corresponding authors on reasonable request.
