# Supplementary material for: Robust Surface-Engineered Tape-Cast and Extrusion Methods to Fabricate Electrically-Conductive Poly(vinylidene fluoride)/Carbon Nanotube Filaments for Corrosion-Resistant 3D Printing Applications
Source: Sci Rep. 2019 Jul 3;9:9618. doi: 10.1038/s41598-019-45992-5 (PMC6610098; doi:10.1038/s41598-019-45992-5)
Supplement: Supplementary file 1 — Supplementary Information [file 41598_2019_45992_MOESM1_ESM.pdf]

Supplementary Information

Robust Surface-Engineered Tape-Cast and Extrusion Methods to Fabricate Electrically-Conductive Poly(vinylidene fluoride)/Carbon Nanotube Filaments for Corrosion-Resistant 3D Printing Applications

Asma Almazrouei[1], Rahmat Agung Susantyoko[2,*], Chieh-Han Wu[2], Ibrahim Mustafa[2], Ayoob Alhammadi[2], and Saif Almheiri[2,3,*]

[*]Corresponding authors:

R. A. Susantyoko (rahmat.a.susantyoko@alum.mit.edu)

S. Almheiri (contact@saifalmheiri.com)

[1] Engineering Systems and Management, Khalifa University of Science and Technology, Masdar Institute, Masdar City, P.O. Box 54224, Abu Dhabi, United Arab Emirates

[2] Department of Mechanical Engineering, Khalifa University of Science and Technology, Masdar Institute, Masdar City, P.O. Box 54224, Abu Dhabi, United Arab Emirates

[3] Mohammed bin Rashid Al Maktoum Solar Park, Dubai Electricity & Water Authority (DEWA), Dubai, United Arab Emirates

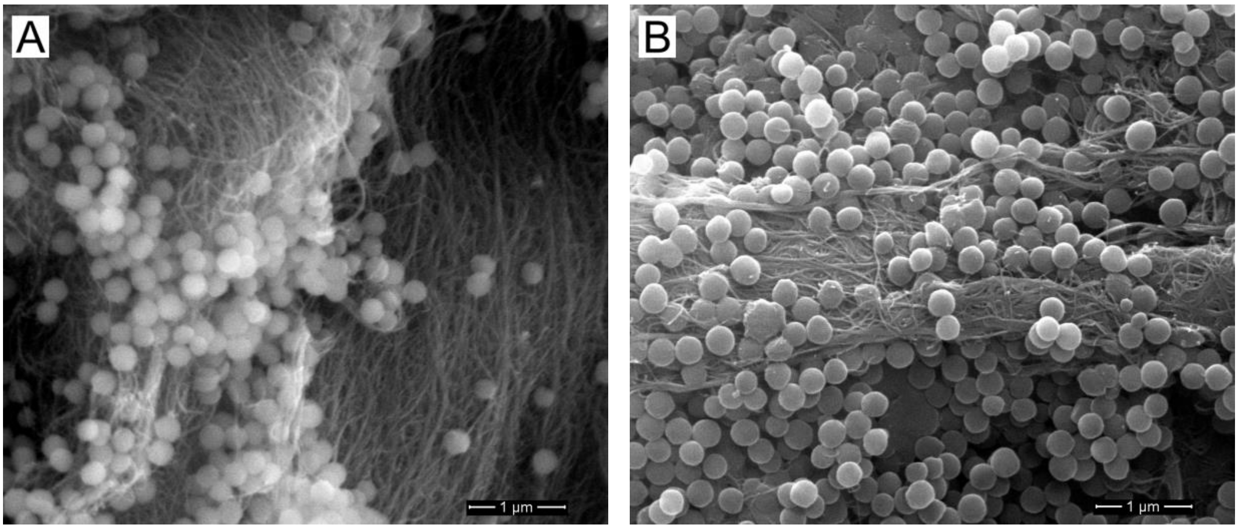

Figure S1: Scanning electron microscopy (SEM) shows the morphology of 90wt%P2-10wt%C1 composite powder (before extrusion) of (A) without ball-milling, and, (B) after ball-milling for 10 minutes.

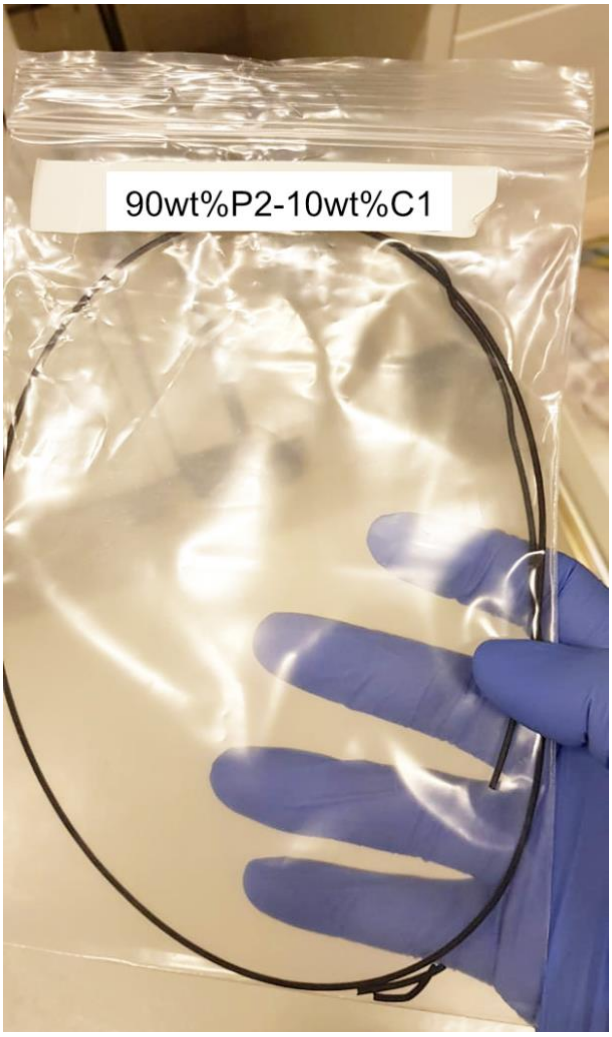

Figure S2: A visual image of the extruded 90wt%P2-10wt%C1 filament

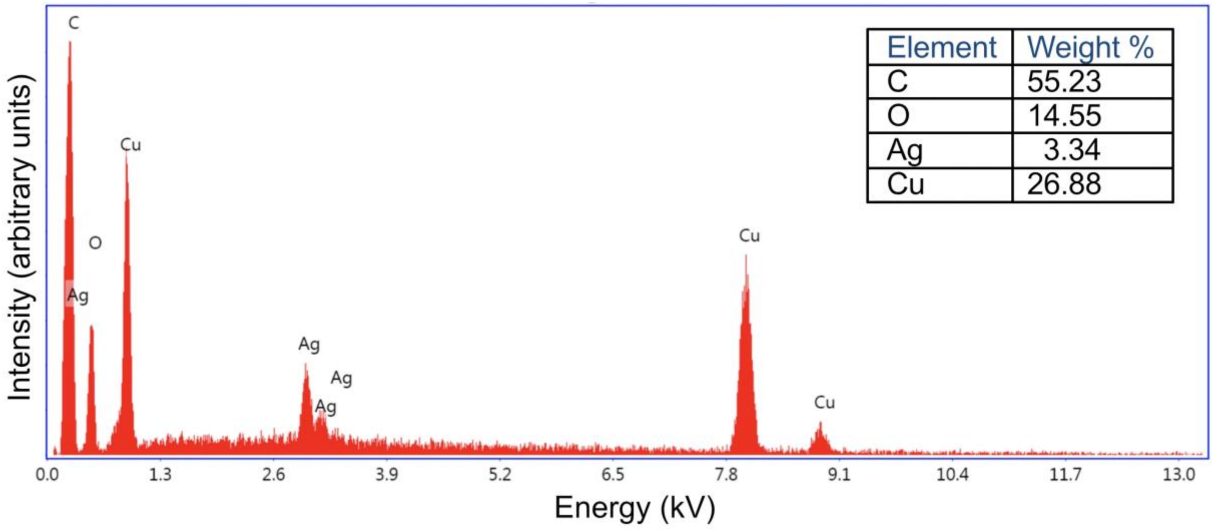

Figure S3: The Energy-Dispersive X-ray Spectroscopy (EDS) of sample F4 (as-received).
